# The Relationship of Health Beliefs on the Stage of Mammography Behavior Adoption amongst Women in Kuantan, Pahang

**DOI:** 10.31557/APJCP.2019.20.6.1913

**Published:** 2019

**Authors:** Hanis Aisyah Ramli, Soo-Foon Moey, Aaina Mardhiah Abdul Mutalib

**Affiliations:** *Department of Diagnostic Imaging and Radiography, Kulliyyah of Allied Health Sciences, International Islamic University Malaysia (IIUM), Pahang, Malaysia. *

**Keywords:** Breast cancer, mammography, health behavior

## Abstract

**Background:**

Breast cancer (BC) awareness is relatively poor among Malaysian women indicated by the presence of BC at a late stage and the low rate of mammography screening. Only a few theoretically based studies have been conducted on Malaysian women’s participation in mammography. Therefore, the objective of this study is to use health belief model (HBM) and stage of change model (SCM) to determine the relationship between health beliefs on the behavioral adoption of mammography amongst women in Kuantan, Pahang.

**Methods:**

Five hundred and twenty women were randomly selected to complete the survey. Data were analyzed using multinomial logistic regression (MLR) to ascertain the multivariate relationships between health beliefs and stage of mammography behavioral adoption.

**Results:**

The MLR test indicates that there is no significant difference in perceived severity, benefits, motivator factors and cues to action between participants in the action stage and the maintenance stage. However, significant differences existed in perceived severity, susceptibility, motivator factors and self-efficacy between the pre-contemplation, relapse and contemplation stage to that of the referenced (maintenance) stage of mammography adoption.

**Conclusion:**

Women in the action stage are more likely to progress towards maintenance stage as they perceived breast cancer as a disease that leads to death and that mammogram screening is beneficial in detecting the disease at an early stage. However, women in the pre-contemplation, relapse and contemplation stage are found unlikely to move towards the maintenance stage as they perceived their risk of getting breast cancer is low.

## Introduction

Breast cancer is the most common cancer amongst women globally and is reported to be the main reason for deaths of cancer amongst women (Ferlay et al., 2010; Jemal et al., 2011). Malaysian women of all ethnic groups in Peninsular Malaysia are also commonly diagnosed with breast cancer (Al-Naggar and Bobryshev, 2012; Tan et al., 2018). Additionally, the prevalence of breast cancer amongst Chinese women is higher as compared to Indians and Malays (Lim et al., 2008). It was found that Chinese women had the lowest breastfeeding rate, shortest breastfeeding duration, lowest parity and late age of full-term pregnancy as compared to Indians and Malays. Thus, these factors contributed to the higher chance of getting breast cancer amongst Chinese women (Yip et al., 2014; Tan et al., 2018). 

Malaysian women were also reported presenting with breast cancer at a younger age in contrast to women from the western countries (Yip et al., 2014). At about 50% of women in Malaysia were found with breast cancer before the age of 50 (Pathy et al., 2011). Similar cancer detection age pattern was reported in India, Taiwan and Singapore (Pathy et al., 2011) in contrast to the American (Jemal et al., 2010) and Dutch (Bastiaannet et al., 2010) women. Further, breast cancer was commonly detected at later stages among Malaysian women as compared to women from the western countries and Singapore (Yip et al., 2014). It was reported that approximately 40% of Malaysian women were detected with breast cancer at stage 3 or stage 4 (National Cancer Registry Report, 2011). 

Early detection is one of the survival determinants from breast cancer which is dependent on disease awareness and also uptake of mammographic screening. Mammography is one of the methods that can diagnose breast cancer at an early stage and is considered as the gold standard for breast cancer screening (Ministry of Health Malaysia Management of breast cancer, 2010; Canadian Task Force on Preventive Health Care, 2011). However, many previous studies found that women’s participation rate in breast cancer screening program was still low (Moodi et al., 2012; Fouladi et al., 2013; Keten et al., 2014; Noroozi et al., 2014). This is possibly owing to the lack of information on program regarding breast cancer screening, lack of knowledge and time, discomfort (Todd and Stuifbergen, 2011), pain, embarrassment, issues of modesty, (Alexandraki and Mooradian, 2010) radiation dose, fear of cancer discovery, fatalism, (Cam and Gumus, 2009) misinformation, irregular check-up, negligence, lack of recommendation from physician and missed screening (Mamdouh et al., 2014). In Malaysia, mammographic screening remains underutilized and is dependent on the women’s initiative to self-refer.

Many studies have been conducted on the subject of breast cancer awareness and breast self-examination in Malaysia (Al-Naggar and Bobryshev, 2012; Yip et al., 2014; Hassan et al., 2015; Mahmud and Aljunid, 2018; Tan et al., 2018). However, only a few research used theoretically based studies in studying Malaysian women in the East Coast of West Malaysia partake in mammographic breast screening behaviors. Therefore, the objective of this study is to use the health belief model (HBM) and stage of change model (SCM) to determine the relationship between health beliefs and the stage of mammography behavioral adoption amongst women in Kuantan, Pahang. With a better understanding of women’s mammography health beliefs and perceptions, it will assist in creating a tailored intervention to encourage women to move towards the advanced stage of mammography such as maintenance stage. It may also provide a baseline assessment for future intervention programs to promote early detection and early management of breast cancer. This study may also help in creating awareness amongst women on the importance of breast cancer screening, hence, increasing the rate of mammogram screening uptake amongst women in Kuantan, Pahang.

**Figure 1:**
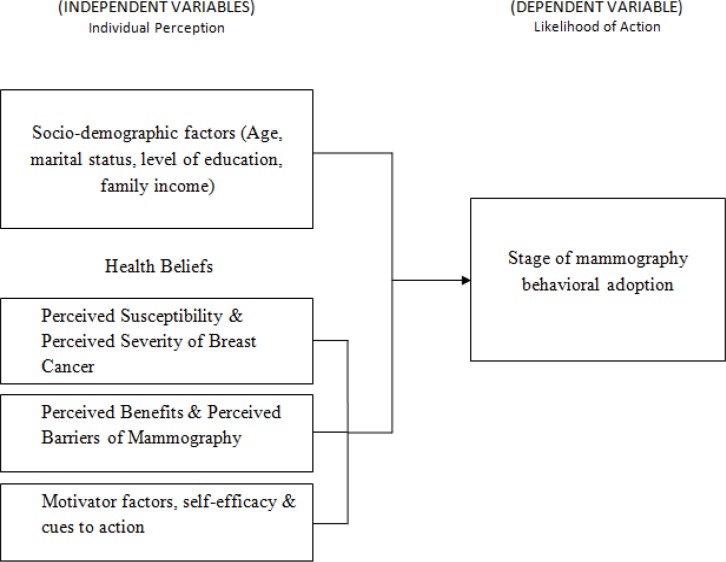
Hypothetical Conceptual Framework of the Study

The Health Belief Model (HBM) was used in the study to predict breast cancer detection behaviors to explain factors influencing mammographic screening behaviors of Malaysian women. It is a psychosocial model that accounts for health behaviors by identifying factors associated with individuals’ beliefs which influence their behaviors (Champion and Scott, 1997). The HBM is derived from the theory that a person behavioral change is primarily based on four factors which are perceived susceptibility, perceived severity, perceived benefits and perceived barriers. Additional factors such as motivator factors, self-efficacy and cues to action were also included in the study. Additionally, the stage of change model (SCM) was used in this study to examine the stages of change a person moves through when adopting the behavior. The SCM proposes that a person moves through a sequence of six stages which are pre-contemplation, relapse, contemplation, relapse risk, action and maintenance (Rakowski et al., 1996). The conceptual framework that governs the study is as in Figure 1.

## Materials and Methods 

*Study design*

A cross-sectional study was used to ascertain the relationship between health beliefs (perceived severity and perceived susceptibility of breast cancer, perceived benefits, barriers of mammography, motivator factors, self-efficacy and cues to action) and the stage of mammography behavioral adoption among women in Kuantan, Pahang.

*Sample size and setting*

A multi-stage sampling method was used to acquire the desired sample size from the population. In the first stage, a cluster sampling method was used to randomly pick three sub-districts in Kuantan. In the second stage, a stratified random sampling was used to randomly pick the polyclinic in the sub-district for the study to be undertaken. Hence, Klinik Kesihatan Balok from Sungai Karang and Klinik Kesihatan Beserah from Beserah were picked. However, as Kuala Kuantan region was larger and more populated than Sungai Karang and Beserah, two polyclinics were randomly picked; Klinik Kesihatan Kuantan and IIUM Family Health Clinic. Using a simple proportion formula for sample size calculation at 5% type 1 error, p<0.05, absolute error at 2%, 520 Malaysians aged between 35 to 70 years, able to read and write in Bahasa Malaysia or English and living in Kuantan were randomly selected for the study. 

*Data collection procedure*

Ethical approvals were obtained from the Kulliyyah Postgraduate Research Center (KPGRC) (approval no: KAHS 173), International Islamic University Malaysia Research Ethics Committee (IREC) (approval no: IREC 2017-075) and Medical Research and Ethics Committee (MREC) (approval no: NMRR-17-2131-37586 (IIR)).

At the health centers, women waiting for Physician consultation were approached individually. An information sheet was used to clarify the intent of the study. Women who verbally agreed to participate were given a set of self-administered questionnaire to complete the survey. The inclusion criteria to participate in the study are female Malaysian citizen aged between 35 to 70 years old and living in Kuantan. The participants must be able to read and write in Bahasa Malaysia or English. Participants that do not meet the fore-mentioned criteria were excluded.

*Instrument*

A set of self-constructed questionnaire was developed from a review of relevant literature of HBM and mammogram and the content was validated by a panel of five health professional experts which includes two professors, one radiologist specializing in diagnosis and screening of breast cancer, an English lecturer and a research scholar in women’s health. The initial questionnaire was further evaluated using exploratory factor analysis from the 103 sets of completed questionnaire obtained via a pilot study. The questionnaire consists of three sections. Section one is pertaining to socio-demographic characteristics (age, race, religion, marital status, level of education, occupation and family income). Section two comprises of 40 questions is to obtain data pertaining to health beliefs of breast cancer and mammogram while section three consists of six questions is to acquire data on the stage of mammography adoption (Appendix A)

*Statistical analysis*

The data were analyzed using the Statistical Package for Social Science (SPSS) version 21.0. Multivariate relationships between dependent variable which is the stage of mammography behavioral adoption and independent variables which are health beliefs (perceived severity, perceived seriousness, perceived benefits, perceived barriers, motivator factors, self efficacy and cues to action) was examined using multinomial logistic regression where maintenance stage was used as the reference stage to compare with the pre-contemplation, relapse, contemplation, relapse risk, and action stages (Rakowski et al., 1996).

## Results

*Demographic characteristics of the respondents*

Table 1 describes the study population. This study sample consisted of 520 respondents, age range from 35 to 70 years old. The respondents had a mean age of 44.64 years (SD = 9.513). Out of 520 respondents, the majority of the respondents were 35 to 40 years old (46.5%) followed by 41 to 45 years old (15.4%), 46 to 50 years old (12.7%), 51 to 55 years old (10.6%), 56 to 60 years old (8.8%), 61 to 65 (3.8%) and 66 to 70 (2.1%). Most of the respondents were married (79.6%) followed by single (13.3%), widow (4.6%) and divorcee (2.5%). Additionally, the majority of the respondents had tertiary education (52.5%) followed by secondary education (41.2%), primary education (6%) and no formal education (0.4%). Most of the respondents had a family income that ranged from Rm3000 to Rm5999 (37.9%), followed by Rm1000 to Rm2999 (33.3%), less than Rm1000 (11.5%), Rm6000 to Rm9999 (10.8%), and more than Rm10000 (6.5%).

**Figure 2:**
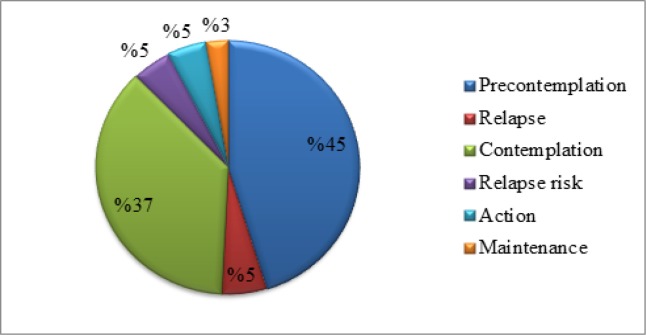
Stage of Behavioral Adoption of Mammography amongst Respondents

The majority of the respondents (n= 235) were at the pre-contemplation stage (45%), followed by the contemplation stage (37%), relapse stage (5%), relapse risk stage (5%), action stage (5%) and maintenance stage (3%), Refer Figure 2.

*Relationship between total health beliefs and stage of mammography behavioral adoption*

Table 2 shows the model fitting information with initial log likelihood value = 900.354 for intercept only model and the final log likelihood value = 23.928 for final model. Meanwhile, the chi-square value obtained is 23.928. The final model can be deduced as better than the intercept only model as the p-value is less than 0.05.

Table 3 reflects the findings of the multivariate relationship between health beliefs model (HBM) as a total and the stage of mammography behavioral adoption amongst respondents. The maintenance stage is used as the reference stage. All stages of behavioral adoption (pre-contemplation, relapse, contemplation, relapse risk, and action) have a significant relationship with total health beliefs (p< 0.05). 

*Relationship between health beliefs and stage of mammography behavioral adoption*

Table 4 indicates the findings of the multivariate relationship between health beliefs model (HBM) and the stage of mammography behavioral adoption amongst respondents in which the maintenance stage is used as the reference stage. The multinomial odds of association between health beliefs (perceived severity, perceived susceptibility, perceived benefits, perceived barriers together with motivator factors, self-efficacy and cues to action) was analyzed using this model. Odds ratio at 95% confidence interval (CI), column Exp. (B) was utilized to explain the relative improvement of mammography behavior for one group compared with the reference group. 

With reference to the maintenance stage, perceived severity (OR = 1.068, CI = 1.005-1.136, p < 0.05), perceived susceptibility (OR = 0.917, CI = 0.870-0.967, p = 0.001), motivator factors (OR= 0.821, CI = 0.712-0.948, p = 0.007) and self-efficacy (OR = 0.899, CI = 0.842-0.960, p = 0.002) was found to have statistically significant relationship with the pre-contemplation stage whilst relapse stage was found to have statistically significant relationship with perceived severity (OR = 1.088, CI = 1.009-1.173, p < 0.05), perceived susceptibility (OR = 0.909, CI = 0.856-0.966, p = 0.002), motivator factors (OR = 0.826, CI = 0.710-0.959, p = 0.012) and self-efficacy (OR = 0.907, CI = 0.844-0.976, p = 0.009). Further, perceived severity (OR = 1.069, CI = 1.006-1.137, p < 0.05), perceived susceptibility (OR = 0.927, CI = 0.880-0.977, p = 0.004), motivator factors (OR = 0.852, CI = 0.738-0.983, p = 0.028), and self-efficacy (OR = 0.894, CI = 0.837-0.955, p = 0.001) also reflected significant relationship with contemplation stage. However, relapse risk stage was found to have statistically significant relationship with only perceived susceptibility (OR = 0.935, CI = 0.880-0.993, p = 0.029). While, perceived susceptibility (OR = 0.934, CI = 0.879-0.992, p = 0.027), perceived barriers (OR = 0.932, CI = 0.873-0.994, p = 0.033) and self-efficacy (OR = 0.925, CI = 0.860-0.996, p = 0.039) indicated significant relationship with action stage of behavioral adoption of mammography (Table 4).

From the model fitting information, the initial log likelihood value = 1,322.233 for intercept only model and the final log likelihood value = 1,232.167 for final model. Meanwhile, the chi-square value obtained is 90.066. The final model can be deduced as better than the intercept only model as the p-value is less than 0.05(Table 5).

## Discussion

In response to the growing concern to explicit construct correlates of HBM to the stage of behavioral adoption of mammography, the results from this study may prove useful in providing detailed information for enhancing health promotion activities in mammography. Although the SCM provides an understanding how health beliefs influence the stage of behavioral adoption, there is a need to find out the differences in the health beliefs at the different stages of adoption of mammography screening behaviors.

In contrast to the HBM and SCM predictions, our results found that there is no significant difference in perceived severity, benefits, motivator factors and cues to action between those in the action stage and the maintenance stage. As women in these stages are already at the advance stages of change, their perception of the seriousness of the disease is similar as they perceived breast cancer as a serious disease that can lead to deleterious consequences such as death. Additionally, it can be seen from the result that women in the action stage are more likely to progress towards maintenance stage as indicated by the odds ratio value (OR = 1.020, CI = 0.952-1.093, p = 0.580). Similarly, previous studies also revealed that those in the action and maintenance stage have significantly higher perceived severity or seriousness of breast cancer (Lindamer et al., 2006). Further, women at these stages of behavioral adoption of mammogram perceived that having a mammogram benefitted them in aiding early detection or monitoring the re-occurrence of breast cancer for women who had been diagnosed with the disease. This finding is concurrent with a previous study (Lindamer et al., 2006) that found those in the action and maintenance stages have high perceived benefits. Results obtained also depict that those in the action stage (OR = 1.058, CI = 0.978-1.145, p = 0.158) are more likely to go to the maintenance stage where they perceived that getting a mammogram done is beneficial for them. In line with the TTM, increasing or maintaining consistent belief in health benefits results in forward stage movement (Menon et al., 2007; Abu-Helalah et al., 2015). Additionally, previous research found that one may need to personally experience mammogram to have high perceived benefits or positive outcomes of health behavior as only being aware of the benefits may not be enough to urge the behavior (Taymoori et al., 2013). This fact is supported by Gierisch et al., (2010) and Taymoori et al., (2013) that found women who have experienced and satisfied with their previous mammogram experiences are more likely to return for another mammogram and maintain doing it in the future. Hence, this explains the behavior of those in the action stage that are more likely to move towards the maintenance stage due to their previous experience. 

Cues to action such as reminder letters, phone calls or text messages would help me to get a mammogram have been reported to have a significant role in helping women to maintain doing mammogram. This is in line with previous studies (Wu and West (2007); Gierisch et al., (2009); Alexandraki and Mooradian, (2010)) found that mammogram reminders are important for women to continue adherence to the mammogram. Further, it was reported that additional support beyond encouragement or cues to action by physicians were also vital for women to sustain doing mammogram over time (Gierisch et al., 2010; Allahverdipour et al., 2011). Consistent with the finding of this study, the results for cues to action shows that those in the action stage (OR = 1.036, CI = 0.968-1.109, p = 0.303) are more likely to move to the maintenance stage.

However, significant differences existed in perceived severity, susceptibility, motivator factors and self-efficacy between the pre-contemplation, relapse and contemplation stage with the maintenance stage of mammography adoption. The results indicated there is a high possibility of women in the pre-contemplation (OR = 1.068, CI = 1.005-1.136, p = 0.034), relapse (OR = 1.088, CI = 1.009-1.173, p = 0.029) and contemplation (OR = 1.069, CI = 1.006-1.137, p = 0.031) stages to move towards the maintenance stage when they perceived that breast cancer is severe (Taymoori et al., 2013). Similar as the women in the action stage of this study, women in the pre-contemplation, relapse and contemplation stages may also perceive the serious impacts of breast cancer on their life and their health status. Hence, the explanation of their behavior to move forward to the maintenance stage. Nevertheless, this study found that women in the relapse stage seemed to retain their beliefs regarding breast cancer and mammography but did not act accordingly (Taymoori et al., 2013).

Further, results from this study revealed that those in the pre-contemplation (OR = 0.917, CI = 0.870-0.967, p = 0.027), relapse (OR = 0.909, CI = 0.856-0.966, p = 0.002), and contemplation (OR = 0.927, CI = 0.880-0.977, p = 0.004) stages are less likely to move towards the maintenance stage indicated by the odds ratio values. This is reflective of the findings of this study which found respondents that were in the pre-contemplation, relapse and contemplation stage have low perceived susceptibility regarding breast cancer which then possibly discouraged them to move to the maintenance stage. Generally, when women do not feel threatened with breast cancer or assumed that their risk of breast cancer is low, they will not undergo a mammogram (Lee et al., 2009; Allahverdipour et al., 2011). This is because they perceived mammogram to be painful, embarrassing (Alexandraki and Mooradian, 2010) and the fear of radiation dose (Cam and Gumus, 2009). These facts are further supported by other studies which discovered that women who are in the action or maintenance stage have higher perceived susceptibility of breast cancer as compared to other mammography behavior adoption stages (Lindamer et al., 2006; Allahverdipour et al., 2011; Shirzadi et al, 2017).

**Table 1:** Demographics of Respondents

	Frequency	Percent	Valid Percent	Cumulative Percent
Age				
35-40	242	46.5	46.5	46.5
41-45	80	15.4	15.4	61.9
46-50	66	12.7	12.7	74.6
51-55	55	10.6	10.6	85.2
56-60	46	8.8	8.8	94
61-65	20	3.8	3.8	97.9
66-70	11	2.1	2.1	100
Total	520	100	100	
Marital status				
Single	69	13.3	13.3	13.3
Married	414	79.6	79.6	92.9
Divorcee	13	2.5	2.5	95.4
Widow	24	4.6	4.6	100
Total	520	100	100	
Level of education				
No formal education	2	0.4	0.4	0.4
Primary education	31	6	6	6.3
Secondary education	214	41.2	41.2	47.5
Tertiary education	273	52.5	52.5	100
Total	520	100	100	
Family income				
<1,000	60	11.5	11.5	11.5
1,000-2,999	173	33.3	33.3	44.8
3,000-5,999	197	37.9	37.9	82.7
6,000-9,999	56	10.8	10.8	93.5
>10,000	34	6.5	6.5	100
Total	520	100	100	

**Table 2:** Model Fitting Information for Relationship between Total Health Beliefs and Stage of Mammography Behavior Adoption

Model	Model Fitting Criteria		Likelihood Ratio Tests	
	-2 Log Likelihood	Chi-Square	df	p-value
Intercept Only	900.354			
Final	876.426	23.928	5	0

**Table 3:** Multivariate Relationship between Total Health Beliefs and the Stage of Mammography Behavioral Adoption amongst Respondents

Stage of Mammography Behavioral Adoption^a^		B	Std. Error	p-value	Exp(B)	95% Confidence Interval for Exp(B)	
						Lower Bound	Upper Bound
Pre-contemplation	Total Health Beliefs	-0.029	0.007	0	0.971	0.957	0.985
Relapse	Total Health Beliefs	-0.024	0.008	0.004	0.976	0.96	0.992
Contemplation	Total Health Beliefs	-0.023	0.007	0.002	0.978	0.964	0.992
Relapse risk	Total Health Beliefs	-0.023	0.009	0.008	0.978	0.961	0.994
Action	Total Health Beliefs	-0.021	0.009	0.016	0.98	0.963	0.996

^a^, reference category: maintenance.

**Table 4:** Multivariate Relationship between Health Beliefs and the Stage of Mammography Behavioral Adoption amongst Respondents

Stage of Mammography Behavioral Adoption^a^		B	Std. Error	p-value	Exp (B)	95% Confidence Interval for Exp(B)	
						Lower Bound	Upper Bound
Pre-contemplation	Perceived Severity	0.066	0.031	0.034	1.068	1.005	1.136
	Perceived Susceptibility	-0.087	0.027	0.001	0.917	0.87	0.967
	Perceived Benefits	0.056	0.033	0.087	1.057	0.992	1.127
	Perceived Barriers	-0.028	0.025	0.253	0.972	0.927	1.02
	Motivator Factors	-0.197	0.073	0.007	0.821	0.712	0.948
	Self-Efficacy	-0.107	0.034	0.002	0.899	0.842	0.96
	Cues to Action	0.038	0.029	0.191	1.039	0.981	1.1
Relapse	Perceived Severity	0.084	0.039	0.029	1.088	1.009	1.173
	Perceived Susceptibility	-0.095	0.031	0.002	0.909	0.856	0.966
	Perceived Benefits	0.062	0.039	0.118	1.064	0.984	1.149
	Perceived Barriers	-0.05	0.031	0.111	0.951	0.895	1.011
	Motivator Factors	-0.192	0.077	0.012	0.826	0.71	0.959
	Self-Efficacy	-0.097	0.037	0.009	0.907	0.844	0.976
	Cues to Action	0.048	0.034	0.16	1.05	0.981	1.123
Contemplation	Perceived Severity	0.067	0.031	0.031	1.069	1.006	1.137
	Perceived Susceptibility	-0.076	0.027	0.004	0.927	0.88	0.977
	Perceived Benefits	0.053	0.033	0.103	1.055	0.989	1.124
	Perceived Barriers	-0.041	0.025	0.093	0.96	0.915	1.007
	Motivator Factors	-0.16	0.073	0.028	0.852	0.738	0.983
	Self-Efficacy	-0.112	0.034	0.001	0.894	0.837	0.955
	Cues to Action	0.052	0.029	0.074	1.054	0.995	1.116
Relapse Risk	Perceived Severity	0.018	0.035	0.618	1.018	0.95	1.091
	Perceived Susceptibility	-0.068	0.031	0.029	0.935	0.88	0.993
	Perceived Benefits	0.072	0.04	0.076	1.074	0.992	1.163
	Perceived Barriers	0.009	0.029	0.764	1.009	0.954	1.067
	Motivator Factors	-0.142	0.078	0.069	0.867	0.744	1.011
	Self-Efficacy	-0.059	0.037	0.108	0.943	0.877	1.013
	Cues to Action	0.009	0.033	0.791	1.009	0.946	1.076
Action	Perceived Severity	0.02	0.035	0.58	1.02	0.952	1.093
	Perceived Susceptibility	-0.068	0.031	0.027	0.934	0.879	0.992
	Perceived Benefits	0.057	0.04	0.158	1.058	0.978	1.145
	Perceived Barriers	-0.071	0.033	0.033	0.932	0.873	0.994
	Motivator Factors	-0.148	0.078	0.057	0.862	0.74	1.005
	Self-Efficacy	-0.077	0.038	0.039	0.925	0.86	0.996
	Cues to Action	0.036	0.035	0.303	1.036	0.968	1.109

^a^, reference category: maintenance.

**Table 5:** Model Fitting Information for Relationship between Individual Health Beliefs and Stage of Mammography Behavior Adoption

Model	Model Fitting Criteria		Likelihood Ratio Tests	
	-2 Log Likelihood	Chi-Square	df	p-value
Intercept Only	1322.233			
Final	1232.167	90.066	35	0

Moreover, the odds ratio values of self-efficacy of respondents in the pre-contemplation (OR = 0.899, CI = 0.842-0.960, p = 0.002), relapse (OR = 0.907, CI = 0.844-0.976, p = 0.009) and contemplation (OR = 0.894, CI = 0.837-0.955, p = 0.001) stages also indicated that they are less likely to move to the maintenance stage. Further, self-efficacy was found to be an important factor in moving women towards the maintenance stage of behavioral adoption of mammography (Menon et al., 2007). This was because women who possess self-efficacy were confident to take the necessary actions to perform the recommended health behavior such as getting a mammogram (Gierisch, 2008). Hence, it can be deduced that women at the lower stages of behavioral adoption such as at the pre-contemplation, relapse and contemplation stage are less likely to get a mammogram because they do not have the self-efficacy for it to be carried out (Russell et al., 2006). Previous study also indicated that women with low self-efficacy and weak behavioral intentions are unable to maintain regular mammogram checkups (Gierisch et al., 2010) as self-efficacy may serve as the behavior intentions’ motivator (Gierisch, 2008). Additionally, strong intentions may act as a motivator factor that triggers planning towards the maintenance stage (Gierisch et al., 2010). Thus, it can be seen that the odds ratio values of motivator factors of those in the pre-contemplation (OR = 0.821, CI = 0.712-0.948, p = 0.007), relapse (OR = 0.826, CI = 0.710-0.959, p = 0.012) and contemplation (OR = 0.852, CI = 0.738-0.983, p = 0.028) stages also indicated that they are less likely to move to the maintenance stage. This is because when women are not motivated to get a mammogram done, they are more likely to stay in the current stage of behavioral adoption. Additionally, previous study also found satisfaction from previous mammogram experience as an important motivator element in encouraging women in maintaining the long-term behavior practices (Gierisch et al., 2010). Thus, the afforded explanations clarify the possible reasons why women in the pre-contemplation, relapse and contemplation stages are less likely to move to the maintenance stage.

In conclusion, the findings of this study indicated that women in the action stage are more likely to move towards the maintenance stage as they perceived breast cancer as a grievous disease and the benefits derived from mammography. Further, women who are at the action stage will continue doing regular mammogram screening if they can be reminded through reminder letter, phone calls or text messages. However, women in the pre-contemplation, relapse and contemplation stage are also more likely to undergo mammogram screening if they can be made to realize breast cancer as a serious disease that can lead to mortality. However, women in these stages are found unlikely to move forward towards the advanced stage of mammography behavior adoption such as the maintenance stage if they perceived they have a low risk of getting breast cancer and not confident or motivated to get a mammogram done.

## Funding Statement

This research was funded by grants from Ministry of Higher Education (MoHE), FRGS17 002-0568. 
